# Transgenerational inheritance of shuffled symbiont communities in the coral *Montipora digitata*

**DOI:** 10.1038/s41598-019-50045-y

**Published:** 2019-09-16

**Authors:** Kate M. Quigley, Bette L. Willis, Carly D. Kenkel

**Affiliations:** 1 Australian Institute of Marine Science, Townsville, QLD Australia; 2 College of Science and Engineering, ARC Centre of Excellence for Coral Reef Studies, James Cook University, Townsville, QLD Australia; 3 Department of Biological Sciences, University of Southern California, Los Angeles, CA USA

**Keywords:** Ecology, Microbiology

## Abstract

Adult organisms may “prime” their offspring for environmental change through a number of genetic and non-genetic mechanisms, termed parental effects. Some coral species may shuffle the proportions of Symbiodiniaceae within their endosymbiotic communities, subsequently altering their thermal tolerance, but it is unclear if shuffled communities are transferred to offspring. We evaluated Symbiodiniaceae community composition in tagged colonies of *Montipora digitata* over two successive annual spawning seasons and the 2016 bleaching event on the Great Barrier Reef. ITS2 amplicon sequencing was applied to four families (four maternal colonies and 10–12 eggs per family) previously sampled and sequenced the year before to characterize shuffling potential in these *M. digitata* colonies and determine if shuffled abundances were preserved in gametes. Symbiont densities and photochemical efficiencies differed significantly among adults in 2016, suggesting differential responses to increased temperatures. Low-abundance (“background”) sequence variants differed more among years than between maternal colonies and offspring. Results indicate that shuffling can occur in a canonically ‘stable’ symbiosis, and that the shuffled community is heritable. Hence, acclimatory changes like shuffling of the Symbiodiniaceae community are not limited to the lifetime of an adult coral and that shuffled communities are inherited across generations in a species with vertical symbiont transmission. Although previously hypothesized, to our knowledge, this is the first evidence that shuffled Symbiodiniaceae communities (at both the inter- and intra- genera level) can be inherited by offspring and supports the hypothesis that shuffling in microbial communities may serve as a mechanism of rapid coral acclimation to changing environmental conditions.

## Introduction

Acclimatization to fluctuating environmental conditions through phenotypic plasticity can promote the persistence of populations and may facilitate subsequent genetic rescue in an era of global climate change^1^. In some cases, plasticity may also be associated with transgenerational effects, whereby the performance of offspring is influenced by environmental conditions experienced by the parents^2^. Inheritance of altered parental phenotypes via transgenerational plasticity may further buffer populations by facilitating acclimatization over generations^3–5^.

The alteration of endosymbiotic Symbiodiniaceae communities has been proposed as an adaptive response to changing environmental conditions (Adaptive Bleaching Hypothesis-ABH)^6–8^. The proportional abundances of Symbiodiniaceae genotypes within adult coral tissues can vary (“shuffle”) to favour those that appear to be more tolerant of prevailing environmental conditions^8–10^. These changes to the Symbiodiniaceae community may have the potential to decrease the predicted frequency of bleaching and the breakdown of the host-symbiont relationship as a result of stress^11^. Shuffling has predominately been described at the inter- genera level (e.g. between *D. trenchii* and *C. goreaui*^8–10,12^) and rarely documented at the intra- genera level^13^. However, it is unclear whether acclimatory changes to the Symbiodiniaceae community are limited to the lifetime of an adult coral or if shuffled communities are inherited across generations in coral species capable of vertical symbiont transmission. The role of microbes in facilitating transgenerational acclimatisation has been discussed (recently termed ‘microbiome-mediated transgenerational acclimatisation’ or MMTA); such a mechanism has yet to be empirically observed, but could facilitate rapid evolution of the coral holobiont in response to environmental change^5,14,15^.

Vertically-transmitting corals provision offspring with Symbiodiniaceae predominantly from the maternal colony^16^. The Symbiodiniaceae community is a heritable trait^17,18^ and genetic constraints (e.g. host controlled immunity and recognition of symbionts) may limit the capacity of vertical transmitters to modify the diversity of their communities compared to those species with environmental acquisition^19,20^ (but see^21^). Evidence for traits other than symbiont shuffling or switching have been found to be transgenerationally inherited. These traits include specificity for particular Symbiodiniaceae genotypes and respiration rates^22,23^. Understanding the capacity for transgenerational acclimation through alterations in maternal Symbiodiniaceae transmission is essential given the major role symbionts play in coral thermal tolerance^9^.

Shuffling often involves a change in the abundance of background (low-abundance) Symbiodiniaceae and potentially affords host corals an improved capacity to increase their thermal tolerance^12,13,24–28^. Symbiodiniaceae from *Durusdinium* (formally clade D^29^), the taxa most often reported (specifically, *D. trenchii*) as instrumental in post-bleaching recovery in corals, normally exist at low levels in coral hosts, even as low as 100 *Durusdinium* cells to 10,000 host cells per square cm^30,31^. Recently, it was found that some colonies of *Acropora millepora* from a naturally cooler population with a ratio of at least 3:1000 *Durusdinium*: *Cladocopium* symbionts were able to shuffle to a *Durusdinium*-dominated community during stress, and subsequently exhibited increased survival and recovery^12^. Shuffling has generally been assessed at the level of genera (*Durusdinium* versus *Cladocopium*). However, an assessment of shuffling at the intra-genera level is essential given the physiological differences at this taxonomic level (e.g. *D. trenchii* versus *C. goreaui*^29^). Background symbionts are functionally important in other host-microbe associations^26,29^. For example, rare members of the bacterial community play disproportionally important roles in nitrogen cycling in their hosts (e.g. *Desulfosporosinus* in peat soil^32^). Therefore, changes in the relative abundances of background Symbiodiniaceae genotypes at the inter- and intra- genera level have the potential to change the host phenotype, thereby contributing to its stress tolerance and recovery^13,25,33^.

We took advantage of a natural thermal stress event, which caused severe bleaching in corals on the Great Barrier Reef (GBR) in 2016 (from 2015–2016)^34^, to evaluate potential symbiont community changes, in particular, shuffling potential, in a vertically-transmitting coral. Using amplicon sequencing of the ITS2 locus, we compared Symbiodiniaceae communities in a sample set comprised of four colonies of *Montipora digitata* and 10–12 eggs from each colony collected during the 2016 mass bleaching event to a replicate sample set collected during the previous summer (2015; identical coral colonies, no thermal anomaly). Although this species is typically dominated by a C15 genotype, it has been shown to host background *Durusdinium* and *Symbiodinium* genotypes^17^. We evaluated the null hypothesis that symbiont communities in these four *M. digitata* are stable and heritable^17^ (H_0_, Fig. S1A) and three possible alternative hypotheses: H_1_- symbiont community composition is stable, but not heritable (no shuffling, no parental effects); H_2_- symbiont community composition can shuffle, and is heritable (shuffling and parental effects); H_3_- symbiont community composition can shuffle, but is not heritable (shuffling, no parental effects).

## Results

### *Montipora digitata* physiology during spawning in a severe bleaching year (2016)

**Figure 1:**
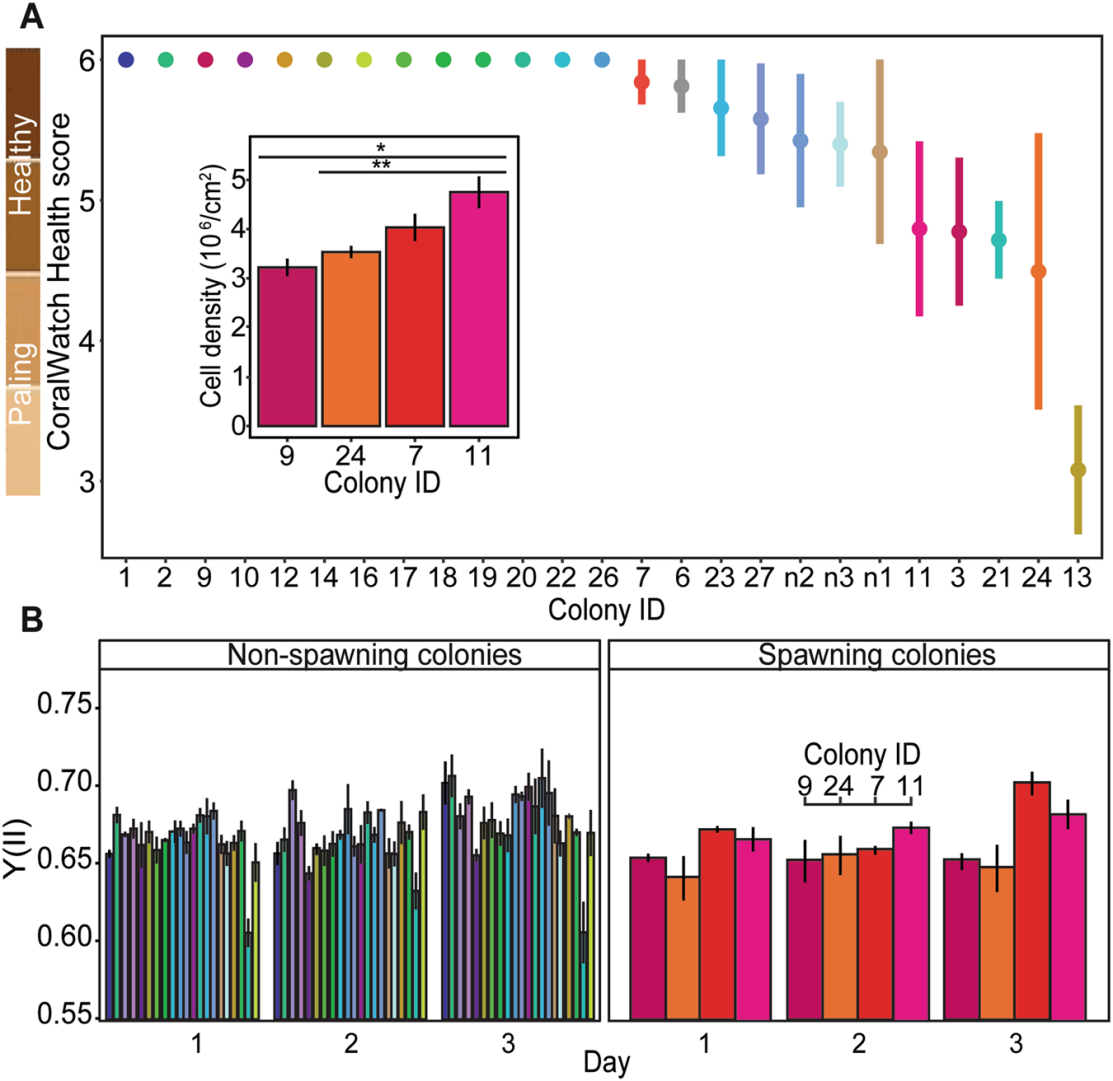
*Montipora digitata* physiological measures during the 2016 bleaching event. (**A**) Bleaching scores (mean CoralWatch Health score ± SEM) per colony during spawning in 2016 in order from no observed bleaching (“healthy”) to paling ( ± SEM = all replicate measures per colony were equivalent). Colors for each colony identity are consistent across all panels, where warm colors designate colonies that spawning in both 2015 and 2016 and cool colors designate colonies that only spawned in 2015. The inset shows mean Symbiodiniaceae cell density ± SEM per colony that spawned in 2016. Asterisks represent significant different treatments after Tukey *posthoc* analysis. Specifically, *p = 0.009, **p = 0.026. (**B**) Effective quantum yield of photosystem II (YII) during the spawning period for non-spawning and spawning colonies.

Twelve of the experimental colonies showed variable signs of paling in 2016, as indicated by the CoralWatch Color Chart (3.08 ± 0.5 to 5.84 ± 0.2). Thirteen colonies did not exhibit any signs of paling or loss of pigmentation (bleaching status = 6) (Fig. 1A). Regardless of this variability in color status across colonies, there was no significant relationship between color and colony identity, spawning status of the colony, or an interaction between the two (identity, spawning status, identity*spawning status: all Negative Binomial Likelihood Ratio Tests Pearson ChiSquared (NB LRT Pr (>Chisq)) >0.9996). Symbiodiniaceae density however varied significantly among the four colonies that spawned (LRT, df = 6, *p* = 0.0097) (Fig. 1 inset), driven by significantly higher densities in colony 11 compared to colonies 9 and 24 (Tukey *posthoc*: *p* = 0.009 and 0.026, respectively).

Overall, effective quantum yield of photosystem II (YII) ranged from 0.7 ± 0.001 (colony n2) down to 0.62 ± 0.009 (colony 21). In general, YII values varied significantly by day, increasing from an average of 0.67 ± 0.002 to 0.69 ± 0.004 in the three days preceding and during spawning (n = 25 colonies with three replicates measured per day; Likelihood Ratio Test-LRT, df = 2, *p* = 1.5e-07, Fig. 1B). Average YII measures also varied significantly among maternal colony (LRT, df = 24, *p* = 2.2e-16), but in a consistent manner through time (no significant interaction between maternal colony identity and day: LRT, df = 48, *p* = 0.127). Spawning colonies had significantly lower YII values compared to non-spawning colonies (0.66 ± 0.02 versus 0.68 ± 0.03; LME, df = 221, *p* = 1e-04). Colony 11 exhibited significantly higher YII values compared to colony 24 (Tukey *posthoc*: *p* = 0.02). Colony 7 also had significantly higher YII values compared to colonies 24 and 9 (Tukey *posthoc*: *p* = 0.006 and 0.028, respectively. YII values did not significantly differ between colonies with the highest cell density (7 and 11, Tukey *posthoc*: *p* = 0.96) or between colonies with the lowest cell density (9 and 24, Tukey *posthoc*: *p* = 0.94).

### ITS2 amplicon sequencing reveals changes in Symbiodiniaceae community composition

On average, more reads per sample were obtained for the 2016 sample set than for the 2015 sample set (32 708 versus 14 020 2 × 250 bp Paired-end reads, respectively), and more reads were retained for the 2016 dataset following quality filtering (78% vs 33%, see Table S2). This resulted in an average of 5 138 reads per sample in 2015 and 26 566 reads per sample in 2016 being available for identification of ITS2 sequence variants.

A total of 34 high confidence variants (those occurring in more than three unique samples) were identified from a subset of 107 high-read abundance samples (z-score >−2.5), representing the four families originating from maternal colonies that spawned in both years (colonies/families 7, 9, 11 and 24). As part of differential abundance testing in the DESeq2 package (see Methods), reads for each of these high confidence variants were variance stabilized, resulting in normalized abundances. A single variant comprised 66% of the normalized read data across samples and a blast search against the GeoSymbio database^35^ identified it as a best match (bit-score = 523) to *Cladocopium* C15. Additional background variants, ranging in abundance from 17% to <1% of the normalized read data across counts also exhibited best matches to C15 (26/34, bit-scores = 507–523) or some derivative thereof (2/34 C15.h, 1/34 C15.6, 1/34 C15.8, 1/34 C15.9, bit-scores = 440–523). The remaining three background sequence variants exhibited best matches to the *Cladocopium* C1 reference (comprising 0.05% of normalized reads, bit-score = 523) and two *Durusdinium* variants (best matches to D1 and D1a = D1–4, *sensu*^36^, bit-scores = 501), comprising 0.04% and 0.014% of normalized reads, respectively.

**Figure 2:**
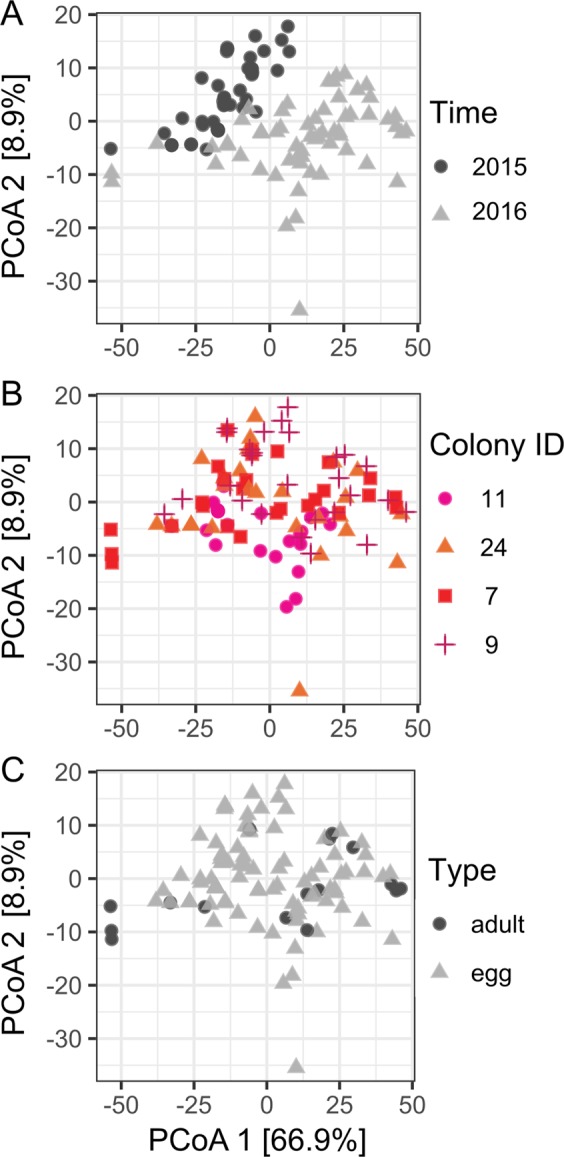
Principal coordinates analysis (PCoA) of log-transformed Manhattan distances among DESeq2 variance normalized symbiont sequence variant counts colored by (**A**) Sampling year: 2015 (non-bleaching), 2016 (bleaching), (**B**) Colony ID and (**C**) Life-stage in *M. digitata*. Colony ID numbers and colors correspond to those in Fig. 1.

Principal coordinates analysis (PCoA) revealed that year was the main factor differentiating symbiont communities among samples by factoring in differences in both the presence/absence of sequence variants and their relative abundances (Fig. 2, see Supplementary Methods). To explore if this pattern could have been driven by the greater quantity of read data generated for the 2016 dataset, the analysis was repeated on a dataset in which high coverage samples from 2016 were randomly subsampled to 7,000 paired-end reads to mimic a reduced sequencing effort. The same major sequence variants were recovered and year remained the main factor differentiating samples (Fig. S2, S3).

**Figure 3:**
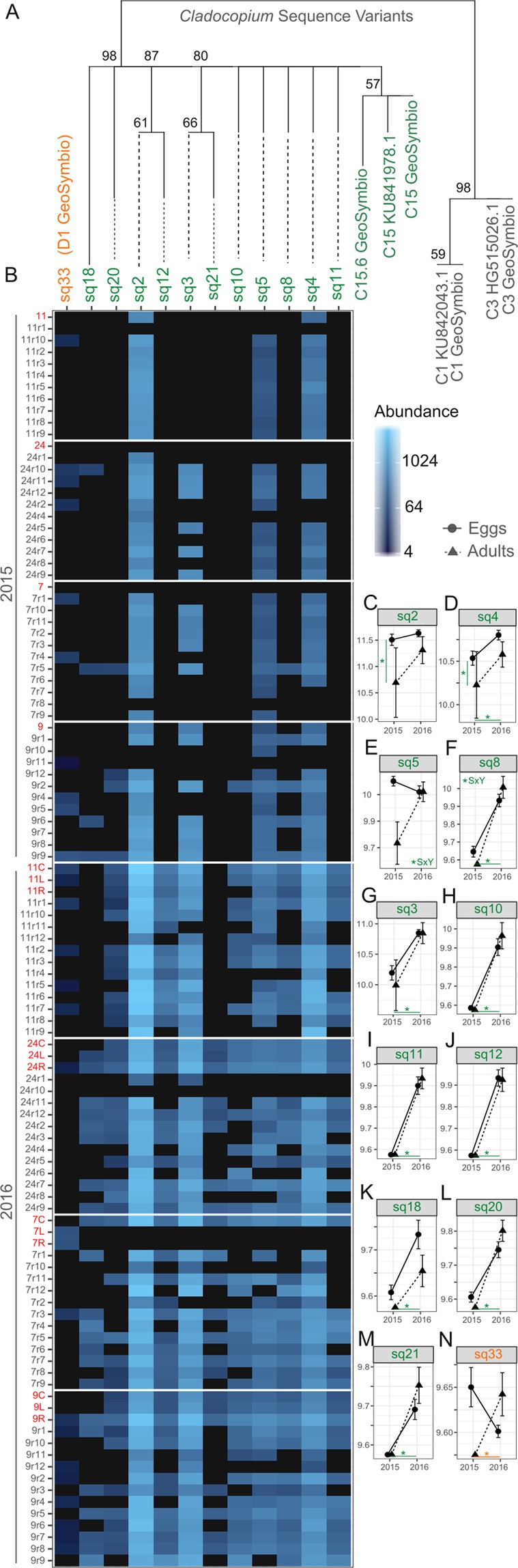
(**A**) Phylogenetic relationships based on Maximum Likelihood among significantly differentially abundant (P_adj_ <0.05) *Cladocopium* (green) and *Durusdinium* (orange) sequence variants between either year and/or life-stages. Reference sequences are derived from either the GeoSymbio database or from NCBI’s nr database. (**B**) Heatmap showing read abundance by sequence variants (top x - axis) and samples (y - axis), grouped by family (numerical grouping) and ordered by year and life-stage (adult samples are listed first and colored red and egg samples are colored grey). (**C**–**N**) Changes in mean abundance for 12 variants between years (x-axis) and by life-stage (E = eggs, A = adults). Mean sequence variant abundance ± SEM for statistically significant factors: (**C**,**D**) P_life-stage_ <0.05,; (**E**,**F**), P_interaction_ <0.05; (**D**, **F**–**N**) P_year_ <0.05. Asterisks across the bottom represent significant differences in abundances across years whereas SxY signifies significant changes across life-stage and years.

A series of generalized linear mixed models performed in the DESeq2 statistical framework further support the dominant effect of year, with twelve of the 34 recovered major sequence variants implicated from the PCoA analysis found to be significantly differentially abundant by year, life-stage or the interaction of the two (Fig. 3, Fig. S1B, S4). Adult colonies shuffled the abundances of 10 sequence variants from 2015 to 2016, with nearly identical changes in seven of these sequences variants also occurring in the eggs of those colonies (Fig. 3). These seven variants, all exhibiting best sequence matches to C15 and comprising 6.6% of total reads, were more abundant in the bleaching year (2016) than in 2015 (P_adj_ <0.05, Fig. 3B, G–M). In addition, while the abundance of sq8 also increased across sampling years, increases were more pronounced in adults (P_year x life stage_ <0.05; Fig. 3F). Sq4 (5% of reads) was also 1.35-fold more abundant in 2016 compared to 2015 (P_adj_ <0.05, Fig. 3D), and showed an additional effect of life-stage, being more abundant in eggs than adults, by 1.27-fold (P_adj_ <0.05).

Only a single sequence variant (sq2) was identified as being significantly differentially abundant between life-stages alone. Sq2, which comprised 17% of the read data across samples, was 1.25-fold more abundant in eggs compared to adults (P_adj_ <0.05, Fig. 3C). Both sq2 and sq4 exhibited a best match to the GeoSymbio *Cladocopium* C15 reference (Fig. 3A), and their abundances were marginally positively correlated with one another across samples (Pearson’s R = 0.29, Fig. S4). In addition, they both exhibited strong negative or neutral relationships with the other *Cladocopium* C15 variants, suggesting that in spite of sequence similarity, they represent a different intragenomic ITS2 variant (Fig. S4).

A D1-variant (sq33), comprising only 0.04% of the total read data, was significantly less abundant in 2016, by 5.25-fold on average, although this pattern primarily appeared to be driven by the decreased abundance in eggs and a concomitant tendency for increased abundance in adults (P_adj_ = 0.014; Fig. 3N). Sq5 increased in abundance in adults across years but decreased in eggs, such that abundances in the two life stages converged in 2016 (P_adj:year x life stage_ <0.05, Fig. 3E).

In addition, abundances of sq3, sq8, sq10, sq11 and sq20 were generally strongly positively correlated across samples, suggesting that they may represent a cluster of intragenomic variants, although a post-clustering curation analysis found insufficient sample-level co-occurrence of amplicon sequence variants to warrant any merging of sequence variants (Supplementary Methods). The largely independent positive relationship found between sequence variants sq18 and sq21 (Pearson’s R = 0.72) may indicate a separate sequence variant grouping (Fig. S4). Given these patterns in abundance and co-occurrence across samples (Figs 3; S4), these sequence variants may represent at a minimum four and at a maximum 11 distinct C15 (Fig. 3C–M) genotypes and one D1 (Fig. 3N) genotype.

### Exploring the potential functional role of Symbiodiniaceae shuffling

Low YII values correlated more strongly with low cell density compared to low coral color scores (R^2^ = 0.62 vs. 0.05, both *p* > 0.21), suggesting that cell density was more important in explaining impaired photosynthetic health (and therefore bleaching related stress) compared to colony color. The parent colony with the highest cell density during the 2016 bleaching event exhibited the greatest relative change in its Symbiodiniaceae community (shuffling score), whereas the Symbiodiniaceae community in the colony with the lowest density during the bleaching event exhibited reduced shuffling (Fig. S5A). Although the sample size was small (n = 4 colonies), a moderate correlation between community change and Symbiodiniaceae cell density was detected, although not significant (R^2^ = 0.33, *p* = 0.42, 95% confidence intervals: 1451143–5618069, Fig. S5B).

## Discussion

This study investigated whether shuffling, quantified as changes in the proportional abundance of Symbiodiniaceae, occurs in a coral species capable of vertical symbiont transmission and whether those changes could be transmitted (e.g. heritable). Although Symbiodiniaceae communities in both adults and eggs of the coral *Montipora digitata* were dominated by one, temporally-stable C15 sequence (66% of read data), variation in multiple, putatively genetically distinct Symbiodiniaceae genotypes found at background abundances occurred across years. Of the 34 sequence variants identified with high confidence, ten showed significant differences in abundance between a bleaching and non-bleaching year, and of these, seven were highly consistent in adults and their corresponding eggs, supporting transgenerational inheritance of Symbiodiniaceae communities that had been shuffled in parental colonies, potentially in response to thermal stress (Fig. S1, H_2_). Fixed differences between life-stages and contrasting changes in adults and eggs further suggest that sequence variants represent distinct Symbiodiniaceae genotypes. In two cases, the abundances of sequence variants in eggs changed across years, but abundances differed independently from communities in adults (H_3_). Whereas the abundances of two sequence variants that likely are another distinct symbiont genotype, differed among life stages, but patterns were stable across years (H_1_) in these four *M. digitata* colonies. Most of the symbiont variation involved the shuffling of diversity within *Cladocopium*, specifically, variants of C15; although we also found evidence of shuffling of *D. trenchii* (Fig. 3). Overall, the most common pattern in background sequences were broad mirroring of shuffled adult communities in eggs of *M. digitata*. Our results suggest that not only is the symbiont community composition able to shuffle in adults of *M. digitata*, but also that shuffling can be heritable.

The importance of shuffling background Symbiodiniaceae for the health of corals is only beginning to be understood. Although some adult corals can shuffle the abundances of Symbiodiniaceae within their endosymbiotic communities in response to temperature changes, thereby increasing the likelihood of survival^26,37^, the implications of transgenerational inheritance of shuffled communities is completely unexplored. In other maternally-inherited symbioses (predominantly bacterial), transmission efficiency of symbionts is influenced by changes in temperature^38,39^. Shuffling (shifts in relative abundances at dominant and background levels) and switching (the secondary acquisition of novel of bacterial symbionts) are both prevalent in insects^40^, and the acquisition of particular symbionts during temperature stress have been shown to provide protective benefits to the holobiont^41^. Furthermore, symbiont-mediated transgenerational effects have been reported to play a role in immune priming in insects^42^ and may be common in plants^43^. Finally, variation in Symbiodiniaceae community composition in coral early life-history stages may serve no functional role in vertical transmitters under non-bleaching conditions (e.g. growth^21^), but remains untested under thermal stress. To our knowledge, we present the first evidence that shuffled proportions of symbiont communities in adult corals are largely preserved in a species which transmits Symbiodiniaceae to its eggs.

Building on previous evidence of the heritability of symbiont transmission in *M. digitata*^17^, our results expand current understanding of the flexibility of the Symbiodiniaceae mutualism in a vertical transmitter by showing that these corals may have greater flexibility to vary their symbiont communities than was previously thought. Early longitudinal studies examining Symbiodiniaceae community composition in vertically-transmitting corals in response to naturally occurring thermal stress events led to the general conclusion that communities were stable^44^ although variation in communities across temperature gradients had been observed^45^. Even when some shuffling was observed among adult colonies, communities eventually reverted to their pre-bleaching abundances following a recovery period^28,46,47^, suggesting that shuffling was temporally limited, although the length of this recovery period might span years (see^13,46^). Changes at the inter- genera level are observed more frequently compared to intra- genera variability. Here we show that intera- genera variability in C15 genotypes can occur; results that corroborate previous evidence of C_I:53 shuffling in response to thermal stress^13^. Compared to *M. digitata*, congeneric adult colonies of *M. capitata* exhibited no interannual changes of symbiont community composition within individual adult corals or their gametes over time^20^. Although the prevalence of shuffling as an environmental response mechanism is still debated, inter- and intra-specific variation has been reported^12,48^. Hence, the stability in *M. capitata* can potentially be attributed to the lack of genetic or environmental variation (i.e. non-bleaching conditions insufficient to elicit shuffling) or shallow sequencing depth (e.g. cloning and subsequent sequencing). Thresholds for shuffling responses may therefore only be brought on by extremely high or fast changes in environmental gradients (e.g. temperature, wave exposure, turbidity^49^). Regardless of the ultimate temporal persistence of such changes in adult corals, we show here that this altered symbiont community can be transgenerationally inherited, although further work is needed to determine if shuffling thresholds for heritable parental effects are similar to those proposed for adults^12^.

Patterns in the changing abundances of background Symbiodiniaceae in *M. digitata* highlight the complexity in the physiological properties conferred by endosymbiotic Symbiodiniaceae on their coral hosts. In horizontal transmitters, the primary pattern described in response to thermal stress so far has been shuffling to increase the abundance of Symbiodiniaceae belonging to *D. trenchii* (represented by changes to D1 and D1–4 sequences in parallel) during bleaching, followed by a decrease in these genotypes once bleaching conditions subside^10,12,24^. Indeed, shifts to a *Durusdinium*-dominated community in adult colonies of *A. millepora* were found to increase thermal tolerance by ~1–1.5 °C^9^. While we observed a trend for a D1 sequence to increase in adult corals, consistent with the increase in thermal stress across years, this sequence variant comprised less than 0.05% of the overall community. Nine sequence variants that were significantly more abundant during the 2016 bleaching year in both adults and their eggs, exhibited a best match to a C15 reference and comprised 12% of the overall community. It has been shown that populations of Symbiodiniaceae with the same ITS2 genotypes can exhibit different thermal tolerances, and in symbiosis can also differentially impact physiological limits of the holobiont^27,50^. Correspondingly, the shuffling response of *M. digitata* colonies to increase the abundance of these different strains of C15 may provide different physiological costs and benefits to their *M. digitata* hosts, which may underpin the differences in abundance. Furthermore, differences in the abundances of two sequence variants, C15 (sq5) and D1 (sq33), which increased in adults but did not show similar patterns in eggs, may reflect differences in costs and benefits conferred to hosts between life-stages, as seen in other invertebrate systems^33^. Prior work in horizontally-transmitting species has suggested that D1 may not always have a thermal protective function in the early-life stages of corals or across species^51^, which may explain why this variant did not increase in relative abundance during the spike in water temperatures. Finally, although read depth was significantly lower for 2015 samples due to the two years being processed on independent sequencing runs, directional changes in abundance and random subsampling to mimic reduced sampling effort both suggest that read depth did not alter the main results. Additional information is therefore needed to elucidate the roles that specific C15 variants and D1 might have in the *M. digitata-*Symbiodiniaceae partnership.

The potential importance of numerically rare background symbionts for the coral-algal symbiosis has long been appreciated^52^, but methods for detecting low-abundance sequence variants have only recently become available^13,53–55^. Consequently, the physiological implications of such shifts are only beginning to be explored. Whilst some background symbionts likely have no notable effect on host fitness^56^, recent network analyses of 46 coral genera provide OTU-based evidence of the importance of cryptic Symbiodiniaceae communities, in which rare symbionts may significantly contribute to symbiosis stability^57^. Similarly, the rare bacterial community is important to holobiont physiology for a range of plant and animal symbioses^32^ and may act to acclimatize the holobiont to new conditions, as postulated for corals^6,10^. Significantly lower Symbiodiniaceae density and YII values indicate that spawning colonies 9 and 24 may have experienced bleaching-level stress. Although YII values of 0.62 may not normally constitute “low” values and therefore not suggest bleaching level stress and photoinhibition, YII values were significantly different to values during non-bleaching years for these colonies. Although the relative changes in YII yields were not large (~11% difference), it is unknown how much change in yields result in negative physiological effects on the host. Although the p-value was not significant, our preliminary finding of a moderate effect size potentially indicates that the adult colony with the largest shifts in Symbiodiniaceae community experienced the lowest levels of bleaching, which suggests that shuffling symbionts could contribute to buffering host physiology under environmental stress. Non-significance may be due to only having four-spawning colonies in conjunction with the large confidence intervals. New metrics may also be needed to better parameterize the extent of shuffling, i.e. to include only dominant symbionts or those at background abundances as well. It is possible that the current parameters do not sufficiently collapse sequence diversity and therefore reflect the changes in community dynamics across years. Further data would be needed to more conclusively demonstrate this relationship.

We also found small but significant differences in photochemical efficiency between spawning and non-spawning colonies, although it is unclear whether the magnitude of these differences is biologically significant. Previous research has presented a link between a coral’s ability to shuffle, buffering of photophysiological performance and spawning success when exposed to cold stress^27^. In this study, photochemical efficiencies remained relatively stable in spawning colonies, whereas non-spawning colonies exhibited larger relative changes, suggesting that symbiont buffering during stress allowed the host to maintain optimal photophysiology and reproductive capacity. Average YII values for spawning colonies may represent smaller deviations away from photophysiological norms, more similar to the non-bleaching year YII values; a phenomenon that has previously been linked to spawning success (“buffered” YII values^27^). Finally, the color-based scores appeared to be uncoupled with Symbiodiniaceae density and photosynthetic health. This potentially suggests that the paling observed in some colonies was due to the loss of photosynthetic pigments per cell, and not a wholesale loss of cells^58^, and is potentially reflected in the spawning colonies having significantly lower YII values. Further work on the long-term physiological and fitness consequences of this heritable shuffling would help to evaluate the potential for this mechanism to facilitate rapid coral acclimation to changing environmental conditions.

## Methods

### Coral spawning and sample collection

*Montipora digitata* is a hermaphroditic, vertically transmitting broadcast spawner^59^. This species primarily spawns one to two days after the full moon in late October to November and again at the end of March, with a gametogenesis of approximately five to six months with no current evidence of partial spawning between months given the length of time needed for egg maturation^60^. Thirty-two colonies of *Montipora digitata* were collected from Hazard (S18°38.069′, E146°29.781′) and Pioneer Bays (S18°36.625′, E146°29.430′) in the Palm Island group on the GBR on the 30^th^ of March and 1^st^ of April 2015. To minimize the collection of clonal colonies, individuals were collected a minimum of 5–10 m apart laterally along the shore given that clones tend to propagate shoreward with wave action^61^ (Heyward, pers. comm). The same colonies were re-collected from Hazard Bay in April 2016 three days before the full moon (five days before spawning), with the exception of colonies 4, 5, 8, 15 and 25, which were presumed dead (Table S1, n2016 = 27). Egg-sperm bundles were collected from all spawning colonies during each year, nine in 2015 (23^rd^ April) and four in 2016 (24^th^ April) (further information on egg/adult tissue collection and preservation in Supplementary Materials).

### Evaluating adult physiology during spawning in 2016 (El Niño year)

To determine the impacts of the El Niño-driven mass thermal stress event on *M. digitata* in 2016, three traits commonly used to evaluate holobiont health were measured: effective quantum yield of photosystem II (YII), colony coloration, and Symbiodiniaceae cell density. YII was measured using Pulse Amplitude Modulated fluorometry (PAM) with a fibre optic cable (diving-PAM, Waltz). PAM measurements were taken at midday (~12:30) one day prior to spawning (22^nd^ April) and two days during the spawning period (23^rd^, 24^th^ April). Three measurements were taken per colony at each time point, with the instrument set at an Intensity of 12, Gain of 4 and F_0_ between 150–300 (Table S1). To test for significant differences in YII due to colony identity or day (as categorical factors), linear mixed models were fit using the package ‘nlme’ in R^62^ (further details on Y(II) model parameterisation in Supplementary Materials).

The presence of a bleaching response was assessed using coral color, Symbiodiniaceae cell density and photosynthetic health. Coral color was quantified by photographing each colony at the time of collection alongside the CoralWatch Color Chart^63^. Derived color values were used as inputs for subsequent generalized linear models (additional details in Supplementary Materials). A negative binomial model (glmer function from ‘lme4’) was used to assess if mean color values varied significantly with colony identity (colony = fixed effect, replicate color chart measures per colony = random effect) (Table S1). Symbiodiniaceae density was determined from spawning colonies using a Neubauer Hemocytometer (Optik Labor, UK) (Table S1) (additional details in Supplementary Materials).

### Symbiodiniaceae community composition analysis

Symbiodiniaceae communities in adults and individual eggs in both 2015 and 2016 were genotyped using paired-end Illumina Miseq amplicon sequencing (2 × 250 bp) of the Internal Transcribed Spacer 2 locus (ITS2)^64^ at the Genome Sequencing and Analysis Facility at the University of Texas at Austin (USA). Samples from all spawning adults (n_2015_ = 9 branches, 1 branch per coral; n_2016_ = 12 branches, 3 branches from each of 4 corals) and 10–12 eggs from each colony were sequenced in two independent runs (corresponding to 2015 versus 2016 samples) (Table S1).

Nine Symbiodiniaceae lineages are currently recognized (A-I), including hundreds of physiologically distinct genotypes within seven formally described genera^29,65^. The multicopy nature of Symbiodiniaceae genomes and the presence of intragenomic variants (repetitive but variable regions) make taxonomic assignments for distinct Symbiodiniaceae sequences difficult^66^. The ITS2 region is a multi-copy, intra-genomically variable maker and thereby results in the detection of multiple sequence variants that are functionally equivalent^67–69^. Discerning between sequences that are accurate representations of distinct functional differences (e.g. different Symbiodiniaceae taxa^29^) or merely intra-genomic variants is pivotal (discussed in^53,55,69^), in which not all sequences represent true unique Symbiodiniaceae genotypes. Given these challenges, one method adopted here is to assign genotypes from amplicon sequence variants involves using a regression approach to determine whether the detection of particular variants are correlated with other variants as a proxy for inferencing inter- versus intra- genomic sequence variants^53,70^. ITS2 sequence variants were inferred using the DADA2 pipeline^70^ in R (v 3.4.1)^71^. DADA2 infers exact sequence variants from amplicon read data, resolving biological differences of even one or two nucleotides and reports fewer false positives than other commonly used OTU-based analysis pipelines^70^. Hence, we are inferring genotypes from amplicon sequence variants (ASVs or simply, variants). Here we designate sequence variants within the genera and genotype level and consider sequence variants from different genera as potential physiologically separate genotypes. Briefly, raw sequencing reads were pre- and post-filtered for quality using error rate models, trimmed, and sequencing variants inferred (full description of bioinformatics steps in Supplementary Methods, including all scripts used to run analyses). These high confidence sequence variants were assigned to the level of genera through a blast search against the GeoSymbio ITS2 database^35^, and the best match was recorded.

We also utilized a post-DADA2 clustering curation step designed to collapse extra diversity of the internal transcribed spacer marker, such as intra-genomic variants, into biologically meaningful units by merging co-occurring, low abundance variants of similar sequence with more abundant ‘parent’ variants^72^. To address the issue of intragenomic variation, we applied a post-clustering curation step using the LULU algorithm^72^. This software collapses extra diversity of the ITS marker, such as intra-genomic variants, by merging variants that are both highly similar in sequence and co-occur across samples, and is analogous to the methodology applied by^73^. However, our sample set contains non-independent biological replicates by design – parents and eggs are expected to host highly similar Symbiodiniaceae sequence communities. Therefore, we only ran LULU on the independent parent coral samples from the 2015 and 2016 collections (2015: n = 9 parent colonies, 2016: n = 3 branches x four parent colonies = 12), with the criteria for merging being an 84% sequence similarity and 70% co-occurrence of amplicon sequence variants across samples. Although variants were highly similar in sequence (Fig. 3), there was a lack of sufficient co-occurrence to warrant any merging and all variants were retained for subsequent analyses.

Additional parent corals and gametes from 2015 were used to validate observed sequence variants (Table S1). Sequence variants were retained if they were identified in at least three unique samples at sufficient abundance (as determined by the z-score statistic, see Supplementary Methods for thresholds on sequence variant determination). However, only the subset of 4 families for which spawning was captured in the two consecutive years was used in the statistical analyses described below to evaluate the impact of life stage and year on symbiont community composition (n_2015_ = 4, n_2016_ = 4). Differential abundance analysis is a helpful framework for statistically evaluating significant differences in abundance across samples, particularly microbial community data or RNA abundance data^74^. This can be implemented in the DESeq2 package^75^ to construct a series of generalized linear models to evaluate differences in the abundance of sequence variants with respect to life stage (adult/egg) and year (2015/2016), including family (7, 9, 11, 24) as a blocking factor. Specifically, this statistical framework uses negative binomial generalized linear models to estimate logarithmic fold changes between treatments by (1) size factors estimation, (2) dispersion estimation, (3) linear model fitting and calculation of Wald statistics^76^.

The MCMC.OTU package^77^ was used to remove sample outliers with low counts overall (z-score <−2.5) and sequence variants that appeared in less than three unique samples prior to statistical analysis. A principal coordinates analysis (PCoA) was performed by computing Manhattan distances of log-transformed normalized variant counts. Manhattan distances when normalized to a sum of 1 are functionally equivalent to Bray-Curtis dissimilarity (specifically ℓ_1_ distance), which incorporates the presence/absence of OTUs as well as their relative abundance^78^. The resulting plots were visualized using the Phyloseq package^79^ to examine the effects of the factors family, year and life stage on Symbiodiniaceae community composition. “Family” is used to designate groups of maternal colonies and their eggs given their high level of genetic relatedness. To determine if sequence variants were correlated with each other, Pearson’s Correlation Coefficients (Pearson’s R^80^) were calculated using the function cor.test(). Deseq2 models were run for 30 iterations and, for models that did not converge, p-values were converted to NAs (‘Not Available’, the R abbreviation for missing data) prior to applying a multiple test correction^81^. The Phyloseq package was used to plot results. Sequence variants of interest were aligned using Clustal Omega^82^, as implemented on the EMBL-EBI web server (https://www.ebi.ac.uk/Tools/msa/clustalo/) and haplotype networks were visualized using the plot functions from the Pegas package^83^. Phylogenetic relationships among significant *Cladocopium* sequence variants were determined with the Phangorn package using Maximum Likelihood^84^. The Hasegawa-Kishino-Yano model of nucleotide substitution including invariant sites^85^ was identified as the best fit model based on AIC, and was used to infer relationships among variants using 100 bootstrap replicates.

To assess the relationship between shuffling and loss of Symbiodiniaceae cells, the composition of the Symbiodiniaceae community was converted to a single quantitative metric and correlated to cell density. Briefly, the single metric was calculated using the Leinster and Cobbold (L-C) diversity metric (^q^D^Z^_ij_(p),^86^) which incorporates variance-normalized abundances, sequence diversity, and rarity of sequence variants (full description and methods in^17^). A shuffle score representing the change in the Symbiodiniaceae community between years was also calculated by subtracting the 2015 diversity score from the 2016 score (Leinster and Cobbold diversity_2016_ - Leinster and Cobbold diversity_2015_). To facilitate comparisons between the two years, only samples collected from the centre of each colony in 2016 were used in order to correspond to samples collected similarly in 2015. Significant sequence variants were calculated using DESeq2 generalized linear models with Benjamini- Hochberg multiple test correction p-values for the factors life stage, year, and family as described above.

## Supplementary information

Electronic Supplementary Material

## Data Availability

All raw sequencing data has been deposited in the NCBI Sequence Read Archive under Accession number SRP077416 and SRP133660.
