# Supplementary material for: Transgenerational inheritance of shuffled symbiont communities in the coral Montipora digitata
Source: Sci Rep. 2019 Sep 16;9:13328. doi: 10.1038/s41598-019-50045-y (PMC6746730; doi:10.1038/s41598-019-50045-y)
Supplement: Supplementary file 1 — Electronic Supplementary Material [file 41598_2019_50045_MOESM1_ESM.docx]

Transgenerational inheritance of shuffled symbiont communities in the coral *Montipora digitata*

Kate M. Quigley, Bette L. Willis, Carly D. Kenkel

**Supplementary materials and methods**

**Coral spawning and sample collection**

Additional colonies outside of the core group of four spawning colonies were collected and assessed for physiological function because we did not know which corals of the original sample set would spawn again in 2016 given temperature stress impacts coral reproductive biology ^1^. In both years, colonies were placed in constant-flow 0.5 µM (2015) or 0.25 µM (2016) filtered seawater in outdoor raceways at Orpheus Island Research Station. Eggs were filtered through a 100 µm mesh and rinsed three times to remove sperm and then individually preserved in 100% ethanol. To minimize stress to adult colonies that might prevent spawning, adult branches were sampled (Table S1, n_2015_ = one branch/colony; n_2016_ = three branches/colony) and preserved in 100% ethanol for genetic analysis of the Symbiodiniaceae communities two days after spawning. Previous re-assessments of colony fecundity post-spawning suggest that all eggs are released during this time ^2^. Therefore, adult tissues would have contained negligible egg tissue.

**Evaluating adult physiology during spawning in 2016 (El Niño year)**

YII represents the percent quanta used by photosystem II of the Symbiodiniaceae cells and is an indication of photosystem functioning and stress during light reactions. Replicate measurers per colony of fluorescence were set as a random effect in the linear mixed model structure. Uniform compound symmetry correlation structure (CorCompSymm) using Day as input values were used to account for autocorrelation issues. Analysis of Deviance Type II tests from the ‘car’ package were used to determine overall main and interactive effects ^3^. The ‘glht’ package was used to extract Tukey p-adjusted values for multiple comparisons from linear models to determine if YII values differed among colonies ^4^. Linear models with the same structure were used to test for significant differences in YII values between spawning and non-spawning colonies. Assumptions of homogeneity of variance, linearity, normality and autocorrelation were checked and tests modified where appropriate.

All images were analysed in ImageJ ^5^ to calculate the average RGB (red, green, blue) brightness values. RGB brightness was calibrated using the CoralWatch chart D1 through D6, where a white/bleached coral would have a chart category of D1 and corresponding brightness value of on average 213.5. Each photo was calibrated to the CoralWatch color chart individually to adjust for differences in exposure, and then three randomly selected RGB brightness values were measured across the whole of each colony. Linear equations were calculated per photo by regressing the D1-D6 standard RGB measurement with their color score (1 - 6). RGB values for each colony were then input into each photo-specific linear equation to derive the color score, which ranged from 2.17 to 6 (where values greater than 6 were designated as 6 ^6^). No deviations in homogeneity of variance, zero-inflation or over dispersion were detected. Likelihood ratio tests (LRT) showed that there was no significant effect of spawning status (spawning or not) or its interaction with dam identity on bleaching status (both Pr (> Chisq) = 1), thus these terms were dropped. LRT and effect sizes were used to assess the overall effect of colony identity on bleaching status (if the means differed significantly across colonies) by comparing the full model to the null model without colony identity using p-values calculated from a chi-squared distribution. Tukey post-hoc tests for cell density and YII values were run using the package “lsmeans” ^7^.

Triplicate cell counts were performed using a ML5000 Brightfield microscope (Meiji Techno) on 450x magnification after nubbins were tissue blasted and standardized to the surface area of each nubbin. Surface areas were calculated using the wax dipping method, using 11 reference standard cylinders ^8^. Only three replicate counts were used given the low variability in replicate cells counts (3.5 – 6.8% standard error of the average counts as seen in figure 1 inset) and much larger margin of error between the bleaching categories (approximate mean difference of 4.8 x 10^6^ cells between bleaching categories ^6^). The margin of error in cell density between replicate counts was therefore approximately an order of magnitude lower (3.8 x 10^5^) than needed to differentiate between bleaching categories.

**Symbiodiniaceae community composition analysis**

Raw fastq reads were pre-filtered by removing any pairs that contained Illumina sequencing adapters (reads with an exact match of at least 12bp) or did not begin with the ITS2 amplicon primer sequence (reads lacking an exact match of at least 10bp), using BBDuk from the BBMap package version 37.75 (http://sourceforge.net/projects/bbmap/).

All analyses were performed in R. The following analysis was completed twice, once for the full dataset, and once for a dataset in which the 2016 reads were randomly subsampled using seqtk (https://github.com/lh3/seqtk) to the mean abundance for the 2015 dataset post-quality filtering (7000 PE reads per sample). Briefly, filtered fastq files were imported into R and read quality profiles were visually inspected for Phred Quality Score Profiles, specifically for those samples with reads falling below a Phred score of 30-35 which denote error rate probabilities ranging from 1:1000 - 1:10,000. Reads were then filtered further, removing reads exhibiting matches to the phiX genome, reads with uncharacterized bases or reads with more than one expected error ^9^. Error rate models use expected error probabilities across the sequence length with quality scores to calculate read accuracy instead of only averaging quality scores per read ^9^. For reads passing these filters, ITS2 amplicon primers were trimmed prior to variant analysis ^9^. Error rate models for forward and reverse reads were run until convergence, and estimated rates were visually inspected to assess goodness of fit with expected error rates which were set to a cut-off of one expected error. Sequence variants were inferred from the entire sequencing dataset using the default options for the “dada” command, which accounts for substitution and indel errors based on learned models, but the “BAND_SIZE” flag was set to 32 as is recommended for ITS data ^9^. Inferred variants were further culled for length (294 - 304 bp accepted based on visual inspection of the sequence variant length histogram and correspondence with the known amplicon size and previous trimming steps) to remove potential products of non-specific priming, and chimeric sequences were removed. In cases where variants matched equally well to multiple GeoSymbio ITS2 references, all top hits were reported.

**Table S1.** Each analysis performed and the number and level of replication for each analysis.

| Year | 2015 | 2016 (bleaching year) |
| --- | --- | --- |
| **Spawning collection** | | |
| N° Collected colonies | 32 individual | 27 of the original 32 colonies, 25 identifiable |
| N° Colonies that spawned | 9 | 4 |
| **Symbiodiniaceae community composition** | | |
| N° of adult branches sequenced | 1 branch/colony for all 9 spawning colonies | 3 branches/colony for all 4 spawning colonies |
| N° of eggs sequenced | 10-11 per colony^1^  Colony 7: 11  Colony 9: 11  Colony 11: 10  Colony 24: 11 | 11-12 per colony^1^  Colony 7: 12  Colony 9: 11  Colony 11: 12  Colony 24: 12 |
| Determination of sequence variants | 9 colonies | 4 colonies |
| Determination of impact of life stage and year on symbiont community composition | 4 colonies (spawned in both 2015 and 2016), 11-12 eggs/ colony | |
| **Physiology during bleaching** | | |
| PAM fluorometry | NA | 3 replicate branches measured per colony |
| ColorWatch color chart | NA | 3 replicate measures for each of 3 replicate branches measured per colony |
| Symbiodiniaceae cell counts | NA | 3 replicate measures per spawning colony |

^1^ Figure 3B shows sequence variant abundance for each egg analysed in each year

**Table S2.** Sequencing output for samples from both 2015 and 2016. All library preparation and sequencing were performed at UT Austin GSAF. Sequences were analyzed used DADA2.

| **Sample Name** | **Raw input** | **Filtered** | **Denoised** | **Merged** | **Tabled** | **Nonchim** |
| --- | --- | --- | --- | --- | --- | --- |
| 2015-M11 | 9424 | 2794 | 2794 | 2494 | 2494 | 2494 |
| 2015-M11r1 | 4362 | 850 | 850 | 583 | 583 | 583 |
| 2015-M11r10 | 14750 | 5705 | 5705 | 5136 | 5136 | 5130 |
| 2015-M11r11 | 3948 | 19 | 19 | 0 | 0 | 0 |
| 2015-M11r2 | 10317 | 3547 | 3547 | 3240 | 3231 | 3227 |
| 2015-M11r3 | 11301 | 4159 | 4159 | 3810 | 3810 | 3808 |
| 2015-M11r4 | 14965 | 5253 | 5253 | 4737 | 4737 | 4736 |
| 2015-M11r5 | 18171 | 6808 | 6808 | 6167 | 6167 | 6166 |
| 2015-M11r6 | 14018 | 4970 | 4970 | 4507 | 4498 | 4497 |
| 2015-M11r7 | 12455 | 4667 | 4667 | 4238 | 4238 | 4236 |
| 2015-M11r8 | 11852 | 4280 | 4280 | 3913 | 3910 | 3909 |
| 2015-M11r9 | 12873 | 5017 | 5017 | 4515 | 4515 | 4513 |
| 2015-M24 | 8949 | 1209 | 1209 | 908 | 908 | 908 |
| 2015-M24r1 | 13978 | 3913 | 3913 | 3398 | 3398 | 3398 |
| 2015-M24r10 | 17867 | 8278 | 8278 | 7620 | 7617 | 7613 |
| 2015-M24r11 | 27812 | 12390 | 12390 | 11253 | 11253 | 11247 |
| 2015-M24r12 | 25649 | 12000 | 12000 | 11072 | 11062 | 11055 |
| 2015-M24r2 | 12775 | 3927 | 3927 | 3437 | 3410 | 3410 |
| 2015-M24r3 | 7226 | 445 | 445 | 333 | 333 | 333 |
| 2015-M24r4 | 8140 | 2761 | 2761 | 2427 | 2407 | 2407 |
| 2015-M24r5 | 13025 | 5756 | 5756 | 5278 | 5278 | 5274 |
| 2015-M24r6 | 13540 | 5942 | 5942 | 5163 | 5154 | 5153 |
| 2015-M24r7 | 23242 | 10971 | 10971 | 10059 | 10059 | 10052 |
| 2015-M24r8 | 10053 | 4474 | 4474 | 3881 | 3881 | 3879 |
| 2015-M24r9 | 22728 | 9538 | 9538 | 8755 | 8755 | 8752 |
| 2015-M26 | 11806 | 4888 | 4888 | 3876 | 3838 | 3838 |
| 2015-M26r1 | 30020 | 12476 | 12476 | 11259 | 11250 | 11236 |
| 2015-M26r10 | 2888 | 251 | 251 | 251 | 251 | 251 |
| 2015-M26r11 | 29533 | 12945 | 12945 | 11947 | 11947 | 11938 |
| 2015-M26r12 | 10299 | 4358 | 4358 | 3963 | 3963 | 3960 |
| 2015-M26r2 | 9273 | 3821 | 3821 | 3814 | 3814 | 3814 |
| 2015-M26r3 | 5398 | 561 | 561 | 437 | 437 | 437 |
| 2015-M26r4 | 4401 | 439 | 439 | 439 | 439 | 439 |
| 2015-M26r5 | 3407 | 311 | 311 | 311 | 311 | 311 |
| 2015-M26r6 | 5576 | 521 | 521 | 408 | 408 | 408 |
| 2015-M26r7 | 2086 | 125 | 125 | 125 | 125 | 125 |
| 2015-M26r8 | 4551 | 464 | 464 | 411 | 411 | 411 |
| 2015-M26r9 | 3357 | 284 | 284 | 284 | 284 | 284 |
| 2015-M28 | 14484 | 5899 | 5899 | 5488 | 5486 | 5486 |
| 2015-M28r1 | 14624 | 6187 | 6187 | 5702 | 5696 | 5692 |
| 2015-M28r10 | 21057 | 5604 | 5604 | 4427 | 4420 | 4420 |
| 2015-M28r11 | 8947 | 2163 | 2163 | 1706 | 1706 | 1706 |
| 2015-M28r12 | 7869 | 118 | 118 | 118 | 118 | 118 |
| 2015-M28r2 | 13122 | 4603 | 4603 | 4079 | 4077 | 4077 |
| 2015-M28r3 | 12792 | 4427 | 4427 | 3911 | 3911 | 3911 |
| 2015-M28r4 | 10889 | 3718 | 3718 | 3341 | 3338 | 3338 |
| 2015-M28r5 | 12607 | 4005 | 4005 | 3191 | 3191 | 3191 |
| 2015-M28r6 | 14502 | 5017 | 5017 | 4039 | 4025 | 4025 |
| 2015-M28r7 | 12361 | 4426 | 4426 | 3788 | 3781 | 3781 |
| 2015-M28r8 | 11349 | 3918 | 3918 | 3130 | 3130 | 3130 |
| 2015-M28r9 | 10940 | 4040 | 4040 | 3585 | 3585 | 3585 |
| 2015-M29 | 13019 | 4660 | 4660 | 3729 | 3701 | 3701 |
| 2015-M29r1 | 11201 | 3544 | 3544 | 2896 | 2872 | 2872 |
| 2015-M29r10 | 7260 | 2704 | 2704 | 2313 | 2308 | 2308 |
| 2015-M29r11 | 6027 | 1963 | 1963 | 1612 | 1612 | 1612 |
| 2015-M29r12 | 3392 | 920 | 920 | 750 | 750 | 750 |
| 2015-M29r2 | 9406 | 3094 | 3094 | 2910 | 2910 | 2910 |
| 2015-M29r3 | 8291 | 2783 | 2783 | 2241 | 2241 | 2241 |
| 2015-M29r4 | 6859 | 1831 | 1831 | 1534 | 1534 | 1534 |
| 2015-M29r5 | 13736 | 4173 | 4173 | 3374 | 3374 | 3374 |
| 2015-M29r6 | 682 | 113 | 113 | 113 | 113 | 113 |
| 2015-M29r7 | 23770 | 9850 | 9850 | 9022 | 9022 | 9021 |
| 2015-M29r8 | 14192 | 2170 | 2170 | 1774 | 1774 | 1774 |
| 2015-M29r9 | 16705 | 5042 | 5042 | 4038 | 4000 | 4000 |
| 2015-M32 | 12008 | 4247 | 4247 | 3645 | 3442 | 3442 |
| 2015-M32r1 | 13299 | 4472 | 4472 | 3867 | 3867 | 3867 |
| 2015-M32r2 | 11316 | 4696 | 4696 | 4347 | 4347 | 4347 |
| 2015-M32r3 | 11610 | 4546 | 4546 | 3904 | 3849 | 3847 |
| 2015-M32r4 | 13144 | 5218 | 5218 | 4533 | 4527 | 4526 |
| 2015-M32r5 | 14179 | 5472 | 5472 | 4999 | 4999 | 4999 |
| 2015-M7 | 8680 | 3739 | 3739 | 3565 | 3356 | 3287 |
| 2015-M7r1 | 12796 | 5404 | 5404 | 4753 | 4753 | 4749 |
| 2015-M7r10 | 25579 | 8734 | 8734 | 8094 | 8094 | 8090 |
| 2015-M7r11 | 14009 | 5192 | 5192 | 4794 | 4794 | 4792 |
| 2015-M7r2 | 14295 | 6025 | 6025 | 5654 | 5654 | 5651 |
| 2015-M7r3 | 15641 | 6981 | 6981 | 6526 | 6526 | 6525 |
| 2015-M7r4 | 25041 | 5986 | 5986 | 5107 | 5102 | 5099 |
| 2015-M7r5 | 26564 | 12019 | 12019 | 11151 | 11128 | 11121 |
| 2015-M7r6 | 13275 | 4796 | 4796 | 4176 | 4176 | 4176 |
| 2015-M7r7 | 14201 | 4307 | 4307 | 3724 | 3724 | 3724 |
| 2015-M7r8 | 11976 | 2718 | 2718 | 2082 | 2082 | 2082 |
| 2015-M7r9 | 13450 | 4491 | 4491 | 3857 | 3838 | 3838 |
| 2015-M8 | 13450 | 4708 | 4708 | 4342 | 4279 | 4110 |
| 2015-M8r1 | 9629 | 3342 | 3342 | 2867 | 2867 | 2867 |
| 2015-M8r10 | 21987 | 9440 | 9440 | 8566 | 8566 | 8563 |
| 2015-M8r11 | 12330 | 4916 | 4916 | 4217 | 4217 | 4213 |
| 2015-M8r2 | 9240 | 3599 | 3599 | 3112 | 3112 | 3112 |
| 2015-M8r3 | 12059 | 4187 | 4187 | 3567 | 3564 | 3561 |
| 2015-M8r4 | 13841 | 5147 | 5147 | 4385 | 4373 | 4371 |
| 2015-M8r5 | 13958 | 5360 | 5360 | 4976 | 4965 | 4960 |
| 2015-M8r6 | 12712 | 4866 | 4866 | 4219 | 4219 | 4215 |
| 2015-M8r7 | 19828 | 6020 | 6020 | 5391 | 5383 | 5382 |
| 2015-M8r8 | 10706 | 4306 | 4306 | 3763 | 3754 | 3754 |
| 2015-M8r9 | 13763 | 5099 | 5099 | 4676 | 4676 | 4675 |
| 2015-M8t | 25380 | 9652 | 9652 | 8433 | 8376 | 8369 |
| 2015-M9 | 12212 | 4541 | 4541 | 4202 | 4202 | 4201 |
| 2015-M9r1 | 27537 | 10603 | 10603 | 9788 | 9748 | 9745 |
| 2015-M9r10 | 16598 | 3826 | 3826 | 2858 | 2835 | 2835 |
| 2015-M9r11 | 9952 | 1456 | 1456 | 1117 | 1117 | 1117 |
| 2015-M9r12 | 20491 | 7809 | 7809 | 6873 | 6801 | 6800 |
| 2015-M9r2 | 43767 | 19213 | 19213 | 17882 | 17829 | 17823 |
| 2015-M9r3 | 472 | 45 | 45 | 45 | 45 | 45 |
| 2015-M9r4 | 13969 | 6297 | 6297 | 5443 | 5439 | 5437 |
| 2015-M9r5 | 15061 | 6483 | 6483 | 5628 | 5628 | 5627 |
| 2015-M9r6 | 31828 | 13683 | 13683 | 12595 | 12573 | 12568 |
| 2015-M9r7 | 34496 | 15066 | 15066 | 13860 | 13850 | 13844 |
| 2015-M9r8 | 27224 | 11998 | 11998 | 10977 | 10977 | 10970 |
| 2015-M9r9 | 34533 | 16113 | 16113 | 14926 | 14872 | 14856 |
| 2016-11C | 64523 | 52775 | 52775 | 52080 | 52057 | 51222 |
| 2016-11L | 36535 | 30178 | 30178 | 29727 | 29718 | 29394 |
| 2016-11R | 24028 | 19910 | 19910 | 19563 | 19559 | 19387 |
| 2016-11r1 | 36850 | 31199 | 31199 | 30945 | 30945 | 30830 |
| 2016-11r10 | 28114 | 23178 | 23178 | 23052 | 23052 | 23037 |
| 2016-11r11 | 21111 | 17678 | 17678 | 17624 | 17624 | 17623 |
| 2016-11r12 | 5498 | 3311 | 3311 | 3259 | 3259 | 3258 |
| 2016-11r2 | 33269 | 27349 | 27349 | 27094 | 27094 | 26867 |
| 2016-11r3 | 37803 | 31700 | 31700 | 31266 | 31253 | 31077 |
| 2016-11r4 | 16958 | 11203 | 11203 | 11017 | 11017 | 10960 |
| 2016-11r5 | 53893 | 44990 | 44990 | 44646 | 44646 | 44491 |
| 2016-11r6 | 48927 | 40820 | 40820 | 40556 | 40556 | 40388 |
| 2016-11r7 | 43625 | 36338 | 36338 | 35912 | 35912 | 35721 |
| 2016-11r8 | 17256 | 11270 | 11270 | 10985 | 10985 | 10980 |
| 2016-11r9 | 59155 | 50862 | 50862 | 50635 | 50635 | 50626 |
| 2016-24C | 32305 | 25283 | 25283 | 24612 | 24588 | 24565 |
| 2016-24L | 33770 | 28462 | 28462 | 28126 | 28126 | 27920 |
| 2016-24R | 54880 | 45008 | 45008 | 44543 | 44512 | 44385 |
| 2016-24r1 | 15390 | 1562 | 1562 | 1487 | 1487 | 1487 |
| 2016-24r10 | 2221 | 1694 | 1694 | 1677 | 1677 | 1677 |
| 2016-24r11 | 32160 | 24495 | 24495 | 24205 | 24205 | 24190 |
| 2016-24r12 | 19209 | 15715 | 15715 | 15305 | 15294 | 15291 |
| 2016-24r2 | 33690 | 28662 | 28662 | 28437 | 28437 | 28435 |
| 2016-24r3 | 33651 | 26662 | 26662 | 26164 | 26164 | 26159 |
| 2016-24r4 | 22683 | 18977 | 18977 | 18880 | 18863 | 18861 |
| 2016-24r5 | 14537 | 9519 | 9519 | 9291 | 9291 | 9288 |
| 2016-24r6 | 33060 | 27925 | 27925 | 27805 | 27805 | 27800 |
| 2016-24r7 | 56084 | 45991 | 45991 | 45555 | 45555 | 45537 |
| 2016-24r8 | 34773 | 28499 | 28499 | 28179 | 28179 | 28071 |
| 2016-24r9 | 28269 | 22613 | 22613 | 22222 | 22222 | 22208 |
| 2016-7C | 68234 | 56854 | 56854 | 56151 | 56049 | 55706 |
| 2016-7L | 59531 | 48745 | 48745 | 47704 | 46538 | 43813 |
| 2016-7R | 60421 | 48497 | 48497 | 47732 | 46415 | 44509 |
| 2016-7r1 | 34508 | 28690 | 28690 | 28357 | 28357 | 28347 |
| 2016-7r10 | 4354 | 2384 | 2384 | 2332 | 2332 | 2332 |
| 2016-7r11 | 33800 | 28320 | 28320 | 28071 | 28071 | 28052 |
| 2016-7r12 | 74412 | 61788 | 61788 | 61245 | 61245 | 61228 |
| 2016-7r2 | 7638 | 5047 | 5047 | 4791 | 4791 | 4790 |
| 2016-7r3 | 45169 | 37309 | 37309 | 36945 | 36945 | 36931 |
| 2016-7r4 | 33939 | 28157 | 28157 | 27899 | 27803 | 27793 |
| 2016-7r5 | 46513 | 38267 | 38267 | 37766 | 37766 | 37737 |
| 2016-7r6 | 17711 | 13106 | 13106 | 12885 | 12885 | 12881 |
| 2016-7r7 | 34223 | 29104 | 29104 | 28743 | 28743 | 28726 |
| 2016-7r8 | 18095 | 14820 | 14820 | 14547 | 14547 | 14541 |
| 2016-7r9 | 24210 | 20465 | 20465 | 20227 | 20227 | 20225 |
| 2016-9C | 16921 | 13002 | 13002 | 12718 | 12662 | 12650 |
| 2016-9L | 21609 | 17803 | 17803 | 17369 | 17352 | 17278 |
| 2016-9R | 73988 | 62981 | 62981 | 62327 | 62215 | 61707 |
| 2016-9r1 | 32509 | 26995 | 26995 | 26643 | 26643 | 26623 |
| 2016-9r10 | 30423 | 25410 | 25410 | 25078 | 25078 | 25066 |
| 2016-9r11 | 6303 | 3891 | 3891 | 3789 | 3789 | 3789 |
| 2016-9r12 | 10492 | 8797 | 8797 | 8738 | 8738 | 8738 |
| 2016-9r2 | 28225 | 23799 | 23799 | 23558 | 23558 | 23554 |
| 2016-9r3 | 11311 | 6811 | 6811 | 6636 | 6636 | 6635 |
| 2016-9r4 | 39351 | 33262 | 33262 | 32971 | 32971 | 32968 |
| 2016-9r5 | 37245 | 30668 | 30668 | 30180 | 30180 | 30169 |
| 2016-9r6 | 27519 | 22754 | 22754 | 22487 | 22448 | 22310 |
| 2016-9r7 | 26541 | 20557 | 20557 | 20241 | 20236 | 20225 |
| 2016-9r8 | 32010 | 26970 | 26970 | 26528 | 26510 | 26494 |
| 2016-9r9 | 31049 | 24865 | 24865 | 24673 | 24673 | 24659 |


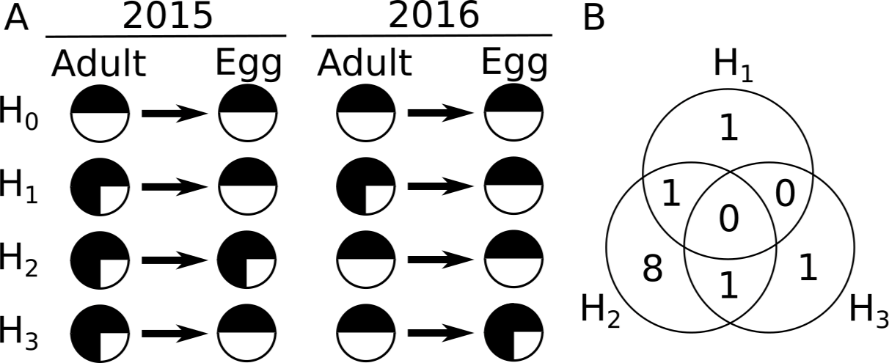


**Figure S1.** (A) Schematic of hypotheses for potential parental effects and/or shuffling across sampling years. (B) Venn diagram showing the number of significantly differentially abundant (P_adj_ < 0.05) Symbiodiniaceae sequence variants by hypothesis.

**Figure S2**. Principal coordinates analysis (PCoA) of log-transformed Manhattan distances among normalized variant counts of sub-sampled dataset (high-yield samples in 2016 randomly sub-sampled to 7000 PE reads) colored by (A) Sampling year, (B) Family and (C) Life-stage.

**Figure S3.** Venn diagram showing the number of significantly differentially abundant

(P_adj_ < 0.05) sequence variants by factor of sub-sampled dataset (high-yield samples in 2016 randomly sub-sampled to 7000 PE reads).


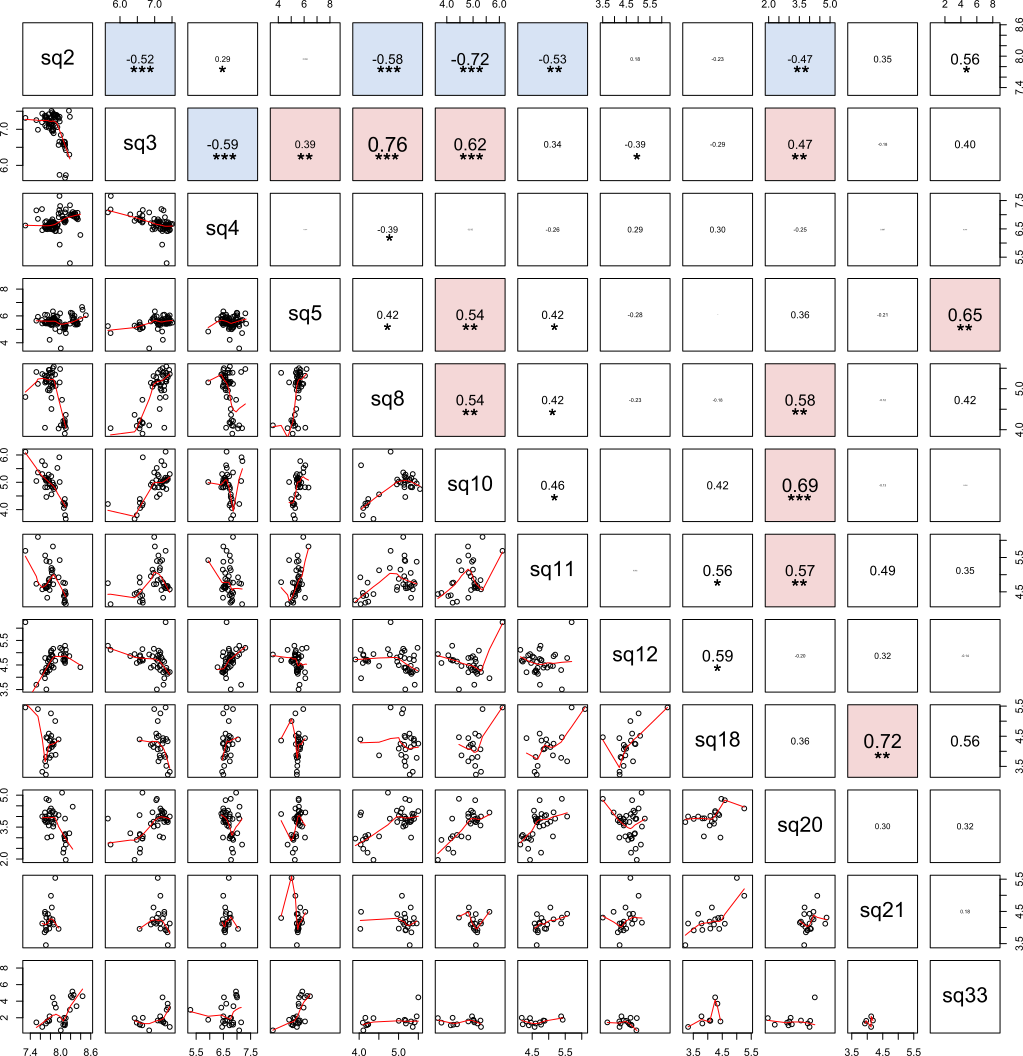


**Figure S4.** Sequence variant abundance correlations across samples. Correlations are Pearson’s Correlation Coefficients (Pearson’s R). Plots are shown below the diagonal, while the corresponding box above the diagonal indicates the magnitude (P-values adjusted for multiple testing, * = P < 0.05, ** = P < 0.01, ***P < 0.001) and direction (red = positive, blue = negative) of the correlation.

**
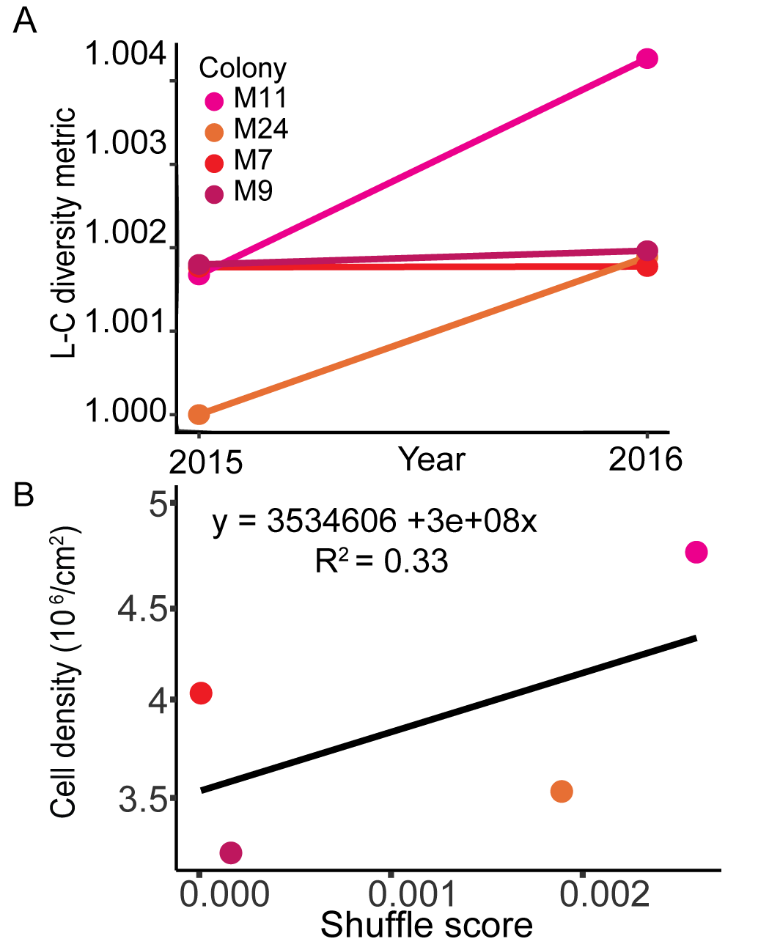
**

**Figure S5.** (A) Symbiodiniaceae community Leinster and Cobbold (L-C) diversity metric in *M. digitata* during non-bleaching (2015) and bleaching (2016) years for each of the four spawning colonies. (B) Relationship between colony cell density scores and the magnitude of shuffling in each spawning colony (R^2^ = 0.33; *p =* 0.42). Low shuffle scores are indicative of communities that did not change between 2015 and 2016, whereas larger shuffle scores are indicative of Symbiodiniaceae communities that did change. Further information on the calculation of the Leinster and Cobbold diversity metric and the shuffle score can be found in the Materials and Methods.

**References**

1. Baird, A. H. & Marshall, P. A. Mortality, growth and reproduction in scleractinian corals following bleaching on the Great Barrier Reef. *Mar. Ecol. Prog. Ser.* **237,** 133–141 (2002).

2. Heyward, A. J. & Collins, J. D. Growth and sexual reproduction in the scleractinian coral Montipora digitata (Dana). *Mar. Freshw. Res.* **36,** 441–446 (1985).

3. Fox, J., Weisberg, S. & Bates, D. car: Companion to Applied Regression. R package version 2.0-2. (2010).

4. Hothorn, T., Bretz, F., Westfall, P. & Heiberger, R. M. multcomp: simultaneous inference for general linear hypotheses, 2008. *URL http//CRAN. R-project. org/package= multcomp. R Packag. version* 0–1 (2008).

5. Rueden, C. T. *et al.* ImageJ2: ImageJ for the next generation of scientific image data. *BMC Bioinformatics* **18,** 529 (2017).

6. Siebeck, U. E., Marshall, N. J., Klüter, A. & Hoegh-Guldberg, O. Monitoring coral bleaching using a colour reference card. *Coral Reefs* **25,** 453–460 (2006).

7. Lenth, R. V & Hervé, M. Package ‘lsmeans’. R package version 2.19. (2015).

8. Veal, C. J., Carmi, M., Fine, M. & Hoegh-Guldberg, O. Increasing the accuracy of surface area estimation using single wax dipping of coral fragments. *Coral Reefs* **29,** 893–897 (2010).

9. Callahan, B. J. *et al.* DADA2: high-resolution sample inference from Illumina amplicon data. *Nat. Methods* **13,** 581–583 (2016).
